# Laparoscopic abdominoperineal resection and myocutaneous flap reconstruction for anal fistula cancer arising from complicated anal fistula: two case reports

**DOI:** 10.1186/s40792-024-02037-y

**Published:** 2024-10-08

**Authors:** Hidemichi Kuroiwa, Yuki Nakamura, Kenji Matsuda, Hiromitsu Iwamoto, Yasuyuki Mitani, Kazuki Shimomura, Norio Takemoto, Toshihiro Sakanaka, Masato Tamiya, Takahiko Hyo, Manabu Kawai

**Affiliations:** Second Department of Surgery, School of Medicine, Wakayama Medical University, 811-1, Kimiidera, Wakayama 641-8510 Japan

**Keywords:** Anal fistula cancer, Myocutaneous flap reconstruction, Complicated anal fistula

## Abstract

**Background:**

Anal fistula cancer is rare and definitive treatment has not yet been established. Laparoscopic abdominoperineal resection is generally the first choice of treatment if the cancer is determined to be resectable. However, complicated anal fistula cancer often requires extensive resection. Using a myocutaneous flap for reconstruction after resection in such cases, radical resection can be performed regardless of the size of the anal fistula cancer.

**Case presentation:**

We report two cases in which we performed laparoscopic abdominoperineal resection with extensive buttock resection and myocutaneous flap reconstruction for widespread anal fistula cancer. One of the cases was reconstruction with a posterior thigh flap, the other was with a bilateral expanded gluteus maximus flap. Both cases were anal fistula cancers that developed from complicated anal fistulas.

**Conclusions:**

If the size of anal fistula cancer is large and extended buttock resection is necessary, radical resection of anal fistula cancer is possible using myocutaneous flap for reconstruction after extended abdominoperineal resection.

## Background

Anal fistula cancer is a rare malignancy which occurs in 0.1% of anal fistulas and occurs with a long-standing perianal fistulas [1, 2]. Patients often present in the advanced stage due to low rate of definitive diagnosis from biopsy [3, 4]. Moreover, for the lack of available data due to their relative rarity, optimal surgical strategy of anal fistula cancer has been not yet established. [5, 6]. Although abdominoperineal resection (APR) is generally standard treatment for anal fistula cancer, it often requires extensive resection to reduce positive margin rate due to larger tumor or surrounding tissue infiltration. Extensive resection for anal fistula cancer requires myocutaneous flap to entail extensive perineal wounds and dead space in the pelvis. However, procedure of myocutaneous flap is invasive and complicated. Here, we report two cases for laparoscopic APR with extensive buttock resection and myocutaneous flap reconstruction for anal fistula cancer arising from complicated anal fistula.

## Case presentation

### Case 1

**Fig. 1:**
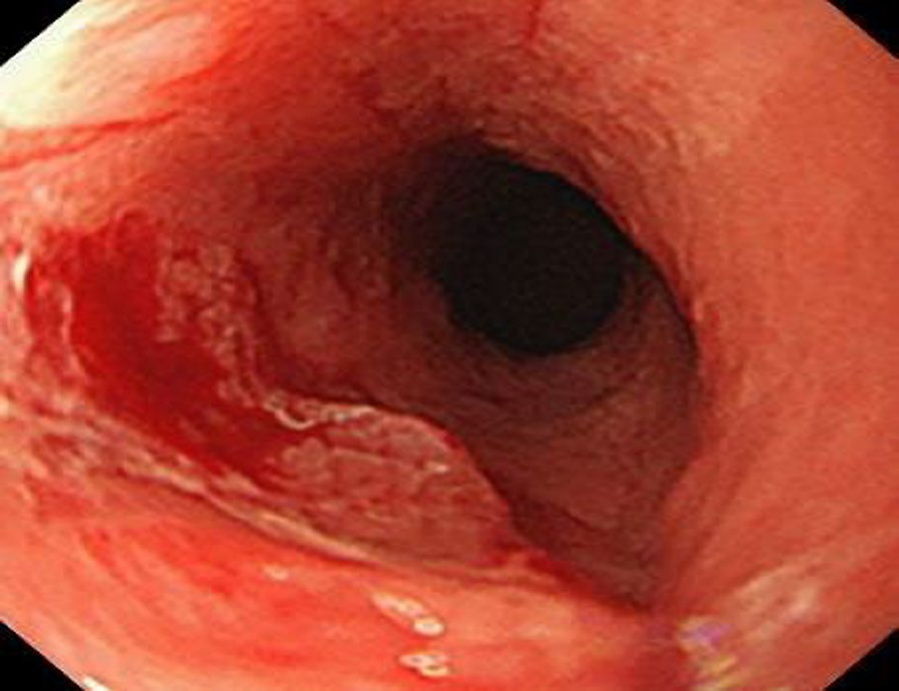
Colonoscopy shows an irregular mucosal surface in the rectal mucosa. Biopsy was performed from the irregular mucosal surface; the result was dysplasia

**Fig. 2:**
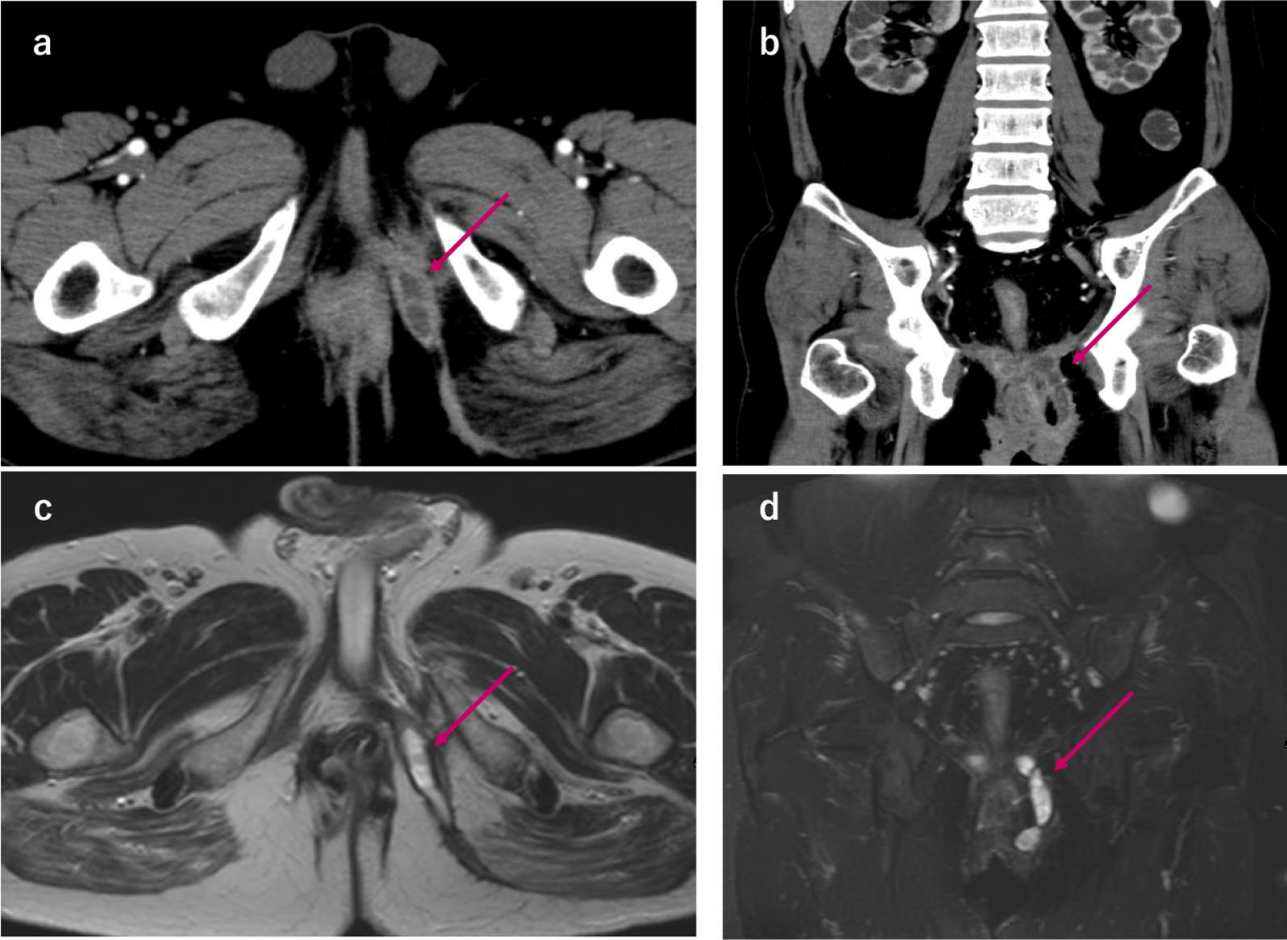
Abdominal CT (**a**, **b**) and MRI (**c**, **d**) show the presence of anal fistula (arrow) to the buttock subcutaneous from the front of sacrum

**Fig. 3:**
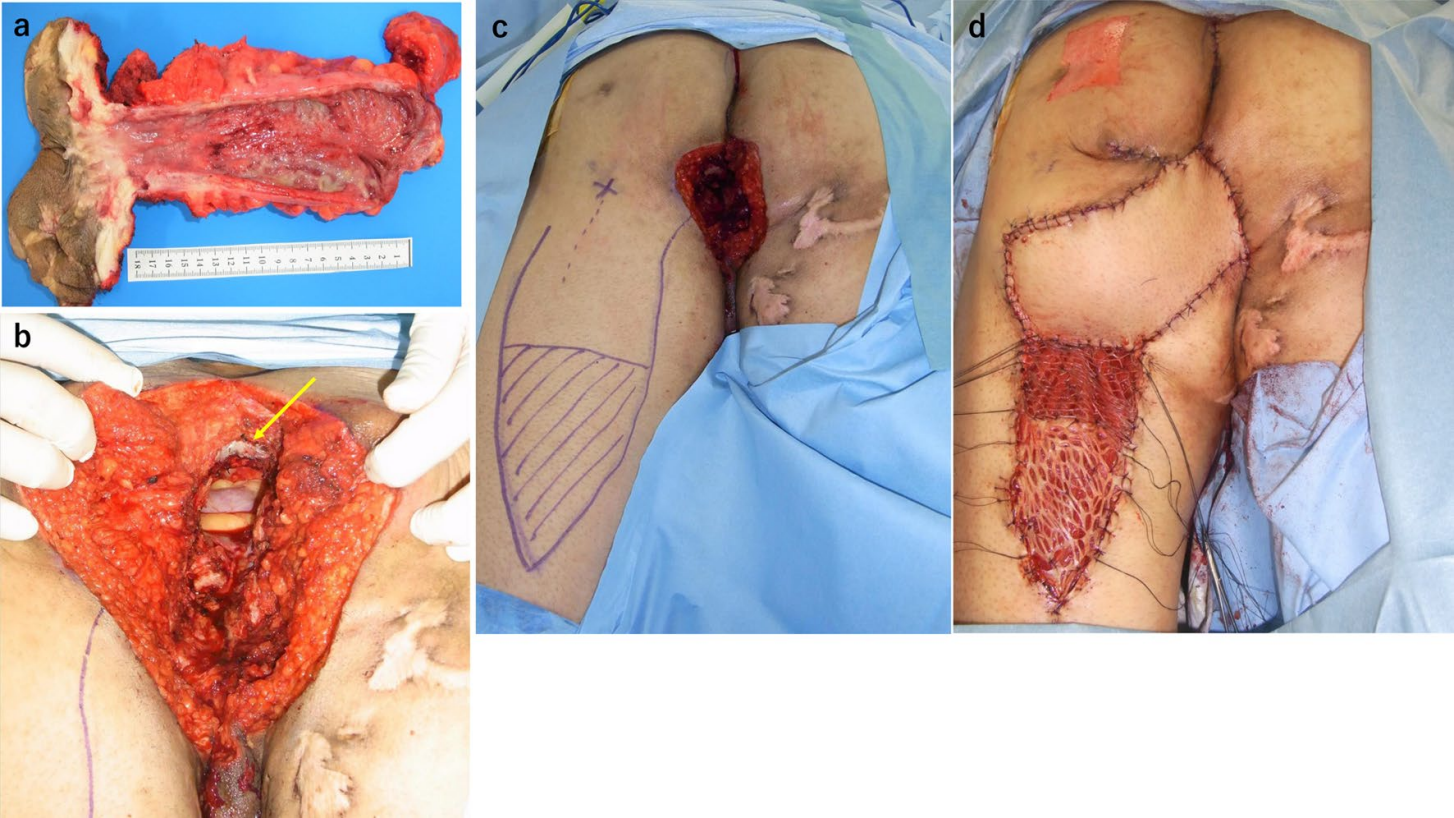
**a** Resected specimen, **b** dead space after resection (arrow: sacrum), **c** design of the myocutaneous flap resection line **d** postoperative wound

**Fig. 4:**
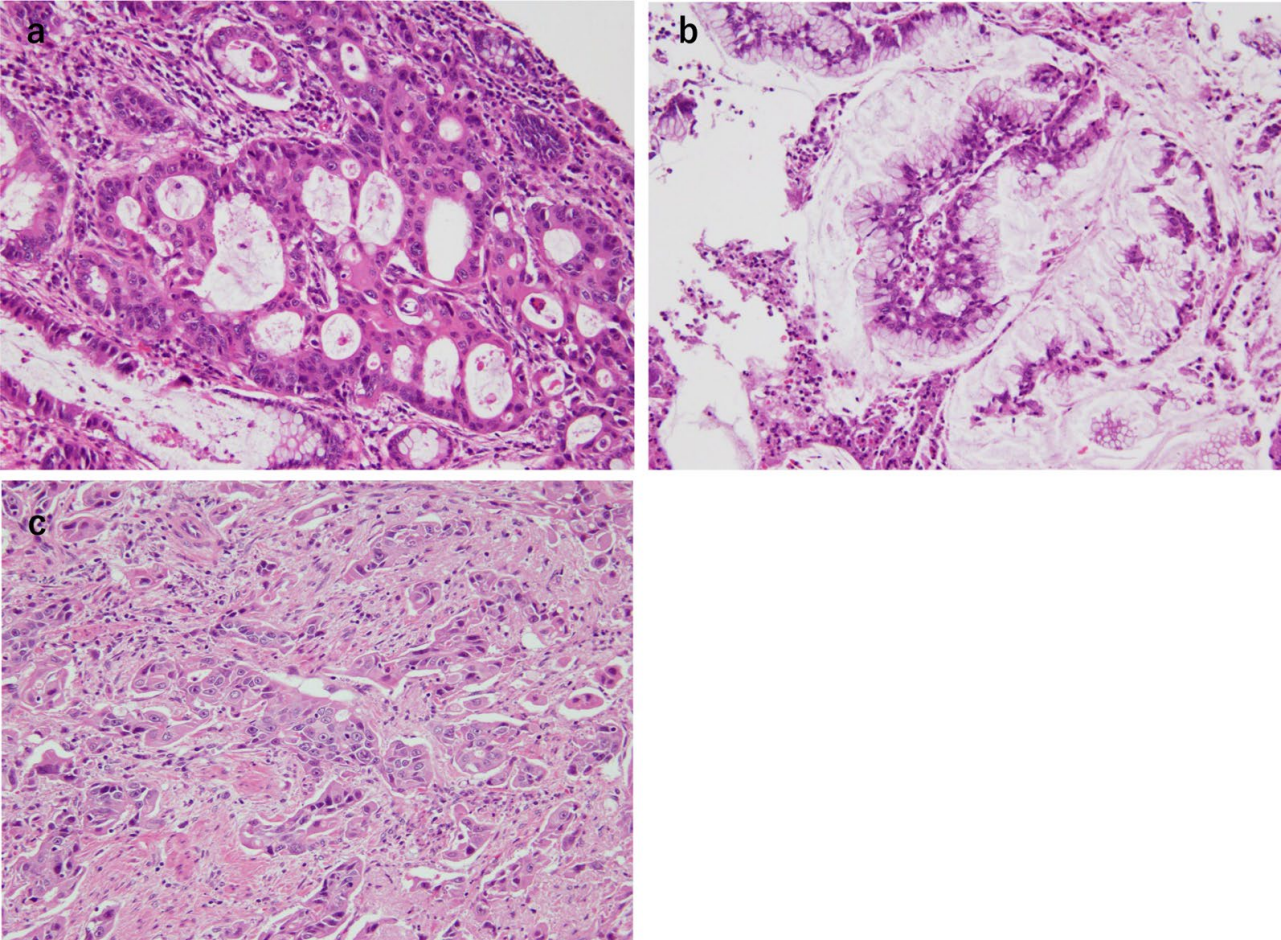
Biopsy of the tumor revealed moderately differentiated tubular adenocarcinoma **a** and mucinous adenocarcinoma **b** and poorly differentiated adenocarcinoma (**c**) (HE × 200)

A 45-year-old man was first diagnosed with Crohn’s disease at 16 years. He had a 22 year history of anal fistula. He was referred to our department for radical treatment of complicated anal fistula at 43 years, having noticed a mucus-like discharge from the anus. Anal fistula cancer was suspected and biopsies were performed from the rectal stricture every 6 months. At 45 years, a biopsy from the vicinity of the primary orifice of the rectal stenosis revealed dysplasia (Fig. 1), and a blood test showed elevated serum carcinoembryonic antigen level in 14.6 ng/ml (normal range ≤ 5.0 ng/ml). Computed tomography (CT) and positron emission tomography (PET) showed abscess formation and accumulation from the front of the sacrum to the subcutaneous area of the buttocks. There was also bilateral inguinal lymph node enlargement. Magnetic resonance imaging (MRI) also showed abscess formation and scarring in the same area (Fig. 2). From these results, we diagnosed anal fistula cancer and decided to perform surgical treatment. The patient underwent laparoscopic APR with extensive buttock resection and sacral resection with posterior thigh flap reconstruction, bilateral lateral lymph node dissection and bilateral inguinal lymph node dissection (Fig. 3). The pathological diagnosis was well-differentiated adenocarcinoma arising from anal fistula. The RAS gene was wild type. No other genetic testing was performed. There was no lymph node metastasis and the tumor was classified as Stage I (T2N0M0) according to TNM classification 8th edition (Fig. 4). However, pathological resection margin was suspected positive (RM1). Eight courses of capecitabine were scheduled as postoperative adjuvant therapy, but the patient developed acute kidney injury from dehydration due to diarrhea, and chemotherapy was discontinued after two courses. Twenty months after surgery, metastasis to bilateral ischial bones and pubic bones was detected by CT and PET. Radiation therapy was performed for these lesions (60 Gy). Four months later, a distant metastatic lesion was found in the bladder. We started chemotherapy with panitumumab monotherapy, but the patient again developed acute kidney injury from dehydration due to diarrhea and stopped chemotherapy after three courses. The patient died 6 months later, 30 months after surgery.

### Case 2

**Fig. 5:**
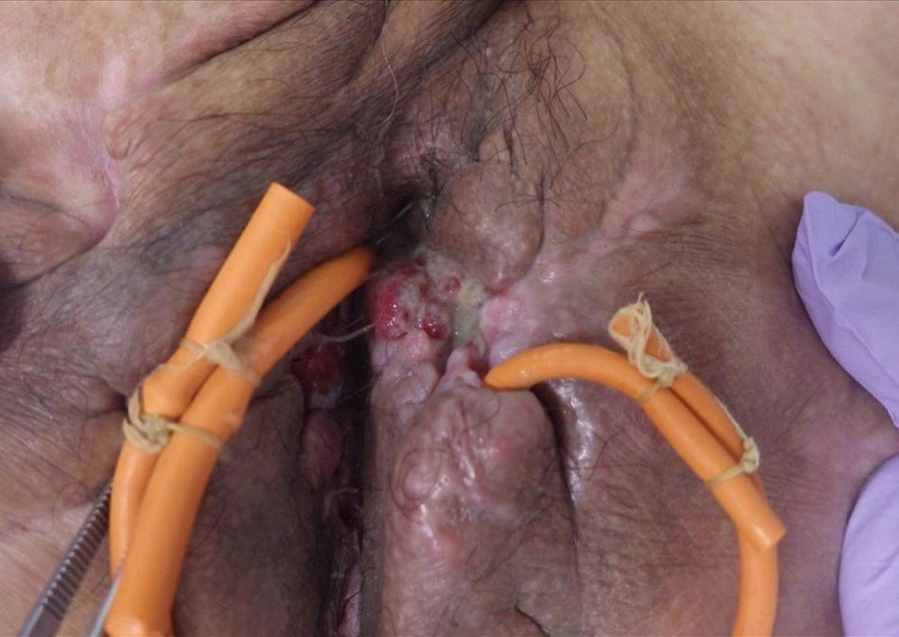
Granulation formation on the left side of the anus. Biopsy revealed well differentiated tubular adenocarcinoma

**Fig. 6:**
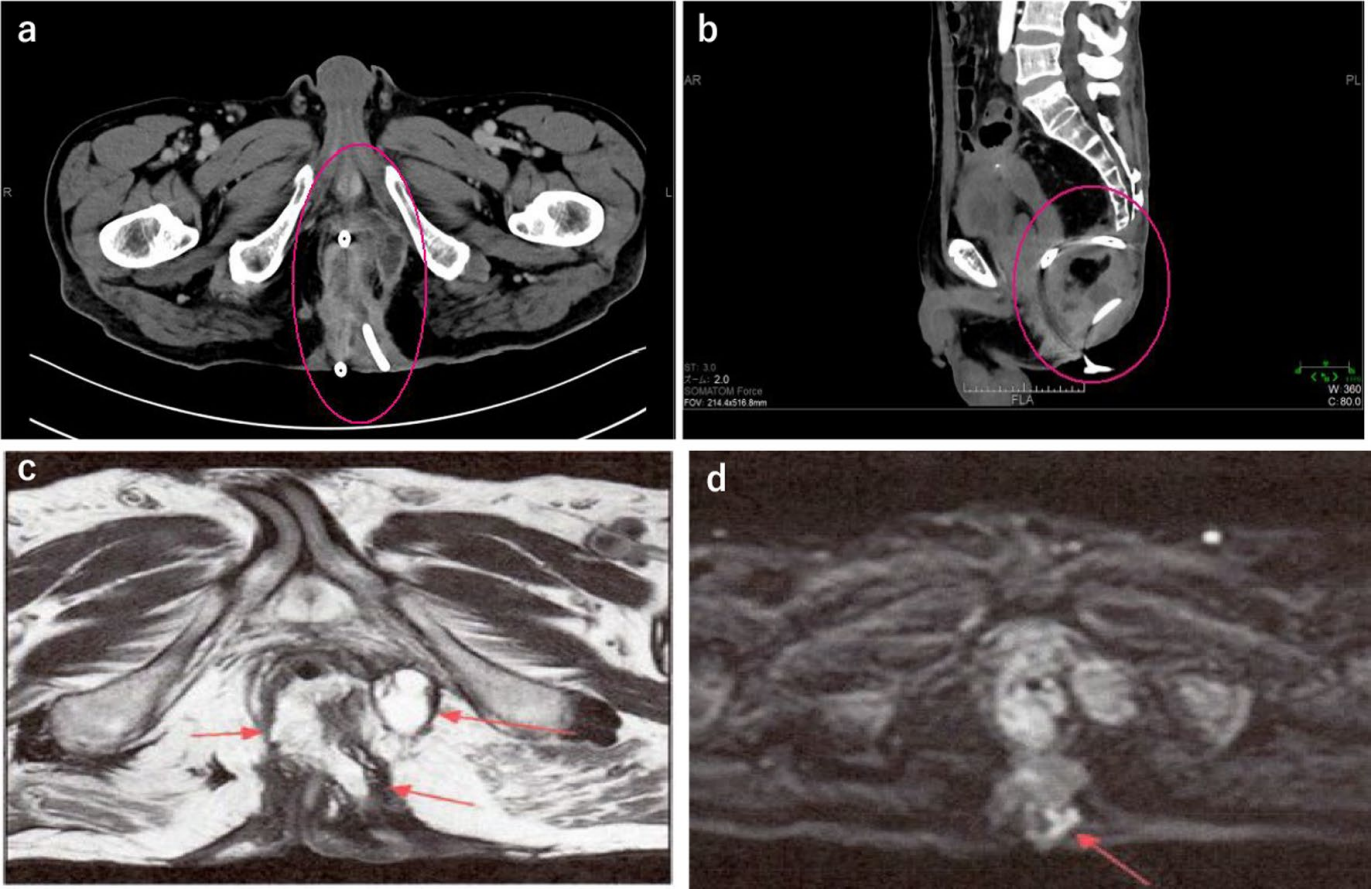
Abdominal CT (**a**, **b**) and MRI (**c**, **d**) shows the presence of anal fistula (circle and arrow) to the lower sacrum from the anus

**Fig. 7:**
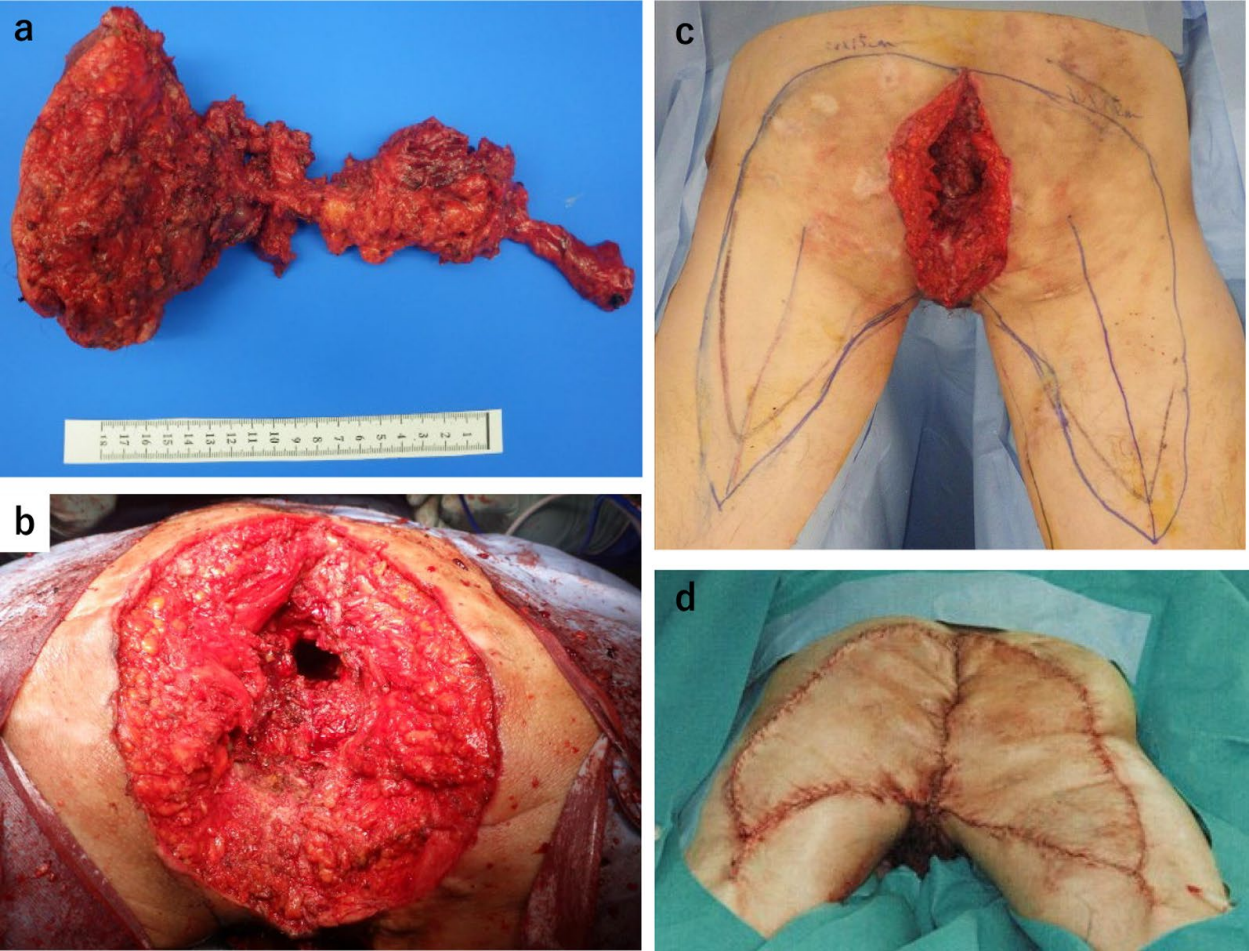
**a** Resected specimen, **b** dead space after resection, **c** design of the myocutaneous flap resection line, **d** postoperative wound

**Fig. 8:**
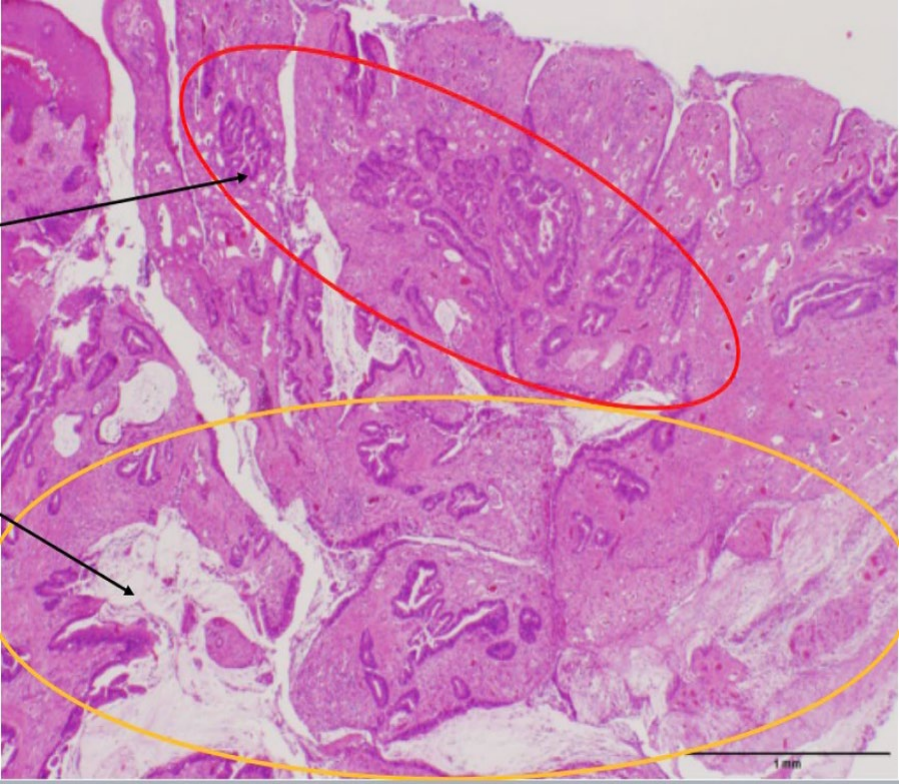
Biopsy of the tumor revealed moderately differentiated adenocarcinoma (red circle) and mucinous adenocarcinoma (yellow circle) (HE × 20)

A 56 year-old man was diagnosed with suspected Crohn’s disease at 31 years. He had 25 year history of anal fistula for which he had repeatedly undergone radical treatment. Granulation formation on the left side of the anus occurred, and a biopsy revealed well differentiated tubular adenocarcinoma (Fig. 5). At this point, he was referred to our department for further investigation and treatment. CT, PET and MRI showed abscess formation and accumulation from the lower sacrum to the anus (Fig. 6). Serum tumor marker levels were increased to 19.7 ng/ml in carcinoembryonic antigen (normal range ≤ 5.0 ng/ml). From these results, the patient was diagnosed with anal fistula cancer and we decided to perform surgery. We performed laparoscopic APR with extensive buttock resection, and bilateral expanded gluteus maximus flap reconstruction (Fig. 7). The pathological diagnosis was moderately differentiated tubular adenocarcinoma. Genetic test results were RAS mutant, BRAF wild type, and microsatellite instability (MSI) negative. There was no lymph node metastasis and the tumor was classified as Stage IIA (T3N0M0) according to TNM classification, 8th edition (Fig. 8). However, pathological findings were suspected to be positive for resection margin (RM1). The patient underwent four courses of capecitabine and oxaliplatin combination therapy as postoperative adjuvant therapy. Six months after the operation, there has been no recurrence.

## Discussion

We report our experience that even extensive anal fistula cancer can be radically resected by reconstruction using an appropriate myocutaneous flap.

Repeated inflammation might predispose the risk factor for development of anal fistula cancer [3]. Increased circulating concentrations of cytokines due to chronic inflammation might influence tumor formation [7]. Patients are usually diagnosed as locally advanced cancer as the rate of definitive diagnosis from a single biopsy tissue is low at 40% [4]. Because the tumor in anal fistula dose not usually pierce the rectal mucosa unless cancer is far advanced. In addition, as the symptom mimic benign inflammatory condition such as perianal oozing or mucinous discharge, the early diagnosis of anal fistula cancer might be missed. Therefore, curative resection for anal fistula cancer requires extensive perineal skin incision and wide perineal tissue excision. APR with the extensive resection and myocutaneous flap reconstruction is often performed for curative resection of anal fistula cancer. Previous case studies have reported about extended APR and myocutaneous flap reconstruction for anal fistula cancer (Table 1) [5, 6, 8–10]. Myocutaneous flap reconstruction might allow wide-ranging extended resection of anal fistula cancer, and then lead to radical resection.

**Table 1:** Anal fistula cancer treated by APR with expanded resection and myocutaneous flap reconstruction in recent literature

Case	Author	Age	Sex	Tumor size (cm)	Surgical method	Flap	Adjubant therapy	Outcomes (mounth)
1	Diaz-Vico T, et al. [5]	66	M	Unknown	APR	VRAM	Capecitabine	28, no recurrence
2	Diaz-Vico T, et al. [5]	71	M	9.5	APR	VRAM, left fasciocutaneous	None	26, recurrence in left inguinal lymph node
3	Diaz-Vico T, et al. [5]	62	M	4.9 × 4.2 × 7.1	APR	VRAM	Capecitabine	19, no recurrence
4	An SH, et al. [6]	67	M	12 × 11 × 9	APR	Bilateral V–Y advancement	5-Fluorouracil	48, no recurrence
5	An SH, et al. [6]	79	M	7 × 4 × 3	APR	None	Unknown	36, no recurrence
6	Chrysikos D, et al. [8]	65	M	3	APR	Gluteus maximus muscle	None	24, no recurrence
7	Ravindran C, et al. [9]	48	M	Unknown	APR	V–Y advancement	Radiation therapy	No recurrence
8	Alsalim A, et al. [10]	43	M	1.8 × 2.7 × 4.1	APR	Myocutaneous pedicled gracilis muscle	Unknown	Unknown
9	Our case	45	M	Unknown	APR with sacral combined resection	Posterior thigh	Capecitabine	20, recurrence in bilateral ischial bones and pubic bones
10	Our case	56	M	8.5 × 8.5	APR	Bilateral expanded gluteus maximus	Capecitabine plus oxaliplatin	6, no recurrence

APR: abdominoperineal resection; VRAM: vertical rectus abdominis myocutaneous flap

We used posterior thigh myocutaneous flap in Case 1 and gluteus maximus myocutaneous flap in Case 2 to repair the perineal defect caused by APR. Optimal myocutaneous flap conditions include good blood flow, close proximity to the postoperative tissue defect, adequate coverage of the defect area, and minimal functional loss due to myocutaneous flap harvesting. Flaps that meet these conditions after APR include the vertical rectus abdominis myocutaneous flap, posterior thigh myocutaneous flap, gluteus maximus myocutaneous flap and gracilis myocutaneous flap, etc. The vertical rectus abdominis myocutaneous flap is widely reported and most used in flap reconstruction after APR [11]. The vertical rectus abdominis myocutaneous flap has the advantage of a long robust pedicle, abundant volume and surface area, ease of harvest while spine position and low incidence of necrosis [12]. However, there are several reasons to limit the use of vertical rectus abdominis myocutaneous flap. These include situations where the rectus abdominis muscle or inferior epigastric vessels have been previously divided, unfavorable abdominal incisions and scarring and the presence of stomas exiting through the rectus abdominis muscle [13]. The posterior thigh flap is robust, easy to raise, and can provide abundant skin and muscle. It does not involve separating the muscles and does not necessarily result in lower limb motor dysfunction, and donor site morbidity is minimal provided direct closure is possible. However, the thigh scars are long and require prone positioning. The posterior thigh flap can be successfully used in the situation to provide both soft-tissue coverage and functional pelvic reconstructions [12, 13]. The gluteus maximus myocutaneous flap is technically easy to raise and provide ample skin and fat. This flap is used mainly for posterior perineal defects but can be extended to reach anterior defects. However, gluteal flaps require prone positioning and usually need to be bilateral [12, 14]. The gracilis myocutaneous flap has the advantage that thin and long muscle can be easily harvested in a spine position, and there is almost no functional sacrifice associated with muscle harvesting. Disadvantages include a lower amount of tissue and a higher risk of flap necrosis compared to the other flaps mentioned above [12]. In Case1, the patient had history of abdominal surgery and the patient underwent laparoscopic APR with extensive buttock resection and sacral resection. The vertical rectus abdominis myocutaneous flap is useful when the perineal defect involves the anterior pelvic compartment but it is not sufficient to fill the posterior pelvic compartments particularly after partial sacretomy [14]. The posterior thigh flap was considered appropriate to fill the large defect with a sufficient amount of skin and muscle. In Case 2, the patient had history of abdominal surgery and had a colostomy. Therefore, flaps arising from the lower extremities were preferable, and considering the size of the defect wound from this surgery, we used expanded gluteus maximus flaps. Regarding postoperative activities of daily living, although both cases felt skin tightness at the surgical site, they were able to walk to the hospital. Our experience with 2 cases shows that there are various options for myocutaneous flap reconstruction used in laparoscopic perineal resection. Therefore, it is necessary to use different myocutaneous flaps depending on their characteristics.

The use of adjuvant therapy is thought to affect patient prognosis; however, there are currently no clear standards for adjuvant therapy. Some case reports in Table 1 have indicated that the prognosis may be improved by adjuvant therapy. We suggest that it is feasible to consider the use of adjuvant therapy, in cases where postoperative adjuvant chemotherapy is deemed suitable. In our department, as with postoperative adjuvant chemotherapy for colorectal cancer, we offer postoperative adjuvant chemotherapy for stage III and high-risk stage II cases. As in the present cases, adjuvant chemotherapy is also administered in cases of suspected positive resection margins.

Although not performed in the present cases, preoperative chemoradiotherapy (CRT) has been reported to be beneficial in ensuring resection margins. Considering the local control effect of preoperative CRT for rectal cancer, preoperative CRT for anal fistula cancer may improve local control and prognosis [15, 16]. In our two cases, though pathological resection margin was suspected positive, a combination of preoperative treatment and extensive surgery could have yielded negative margins.

## Conclusion

If the size of anal fistula cancer is large and extended buttock resection is necessary, radical resection of anal fistula cancer is possible using myocutaneous flap for reconstruction after extended APR.

## Data Availability

Data will be made available on reasonable request.

## Abbreviations

APR: Abdominoperineal resection
CT: Computed tomography
PET: Positron emission tomography
MRI: Magnetic resonance imaging
MSI: Microsatellite instability
CRT: Chemoradiotherapy
